# Protective Effects and Mechanism of *Meretrix meretrix* Oligopeptides against Nonalcoholic Fatty Liver Disease

**DOI:** 10.3390/md15020031

**Published:** 2017-02-14

**Authors:** Fangfang Huang, Shasha Zhao, Fangmiao Yu, Zuisu Yang, Guofang Ding

**Affiliations:** School of Food Science and Pharmacy, Zhejiang Provincial Engineering Technology Research Center of Marine Biomedical Products, Zhejiang Ocean University, Zhoushan 316000, China; gracegang123@126.com (F.H.); zhaoshasha1216@126.com (S.Z.); fmyu@zjou.edu.cn (F.Y.)

**Keywords:** NAFLD, mitochondrial membrane potential, apoptosis, JNK

## Abstract

*Meretrix meretrix* oligopeptides (MMO) derived from shellfish have important medicinal properties. We previously obtained MMO from alcalase by hydrolysis processes. Here we examine the protective effects of MMO against nonalcoholic fatty liver disease (NAFLD) and explored the underlying mechanism. Human Chang liver cells were used in our experiments after exposure to palmitic acid at a final concentration of 15 μg/mL for 48 h to induce an overload of fatty acid as NAFLD model cells. Treatment with MMO for 24 h increased the viability of the NAFLD model cells by inhibiting apoptosis. MMO alleviated oxidative stress in the NAFLD model cells by preserving reactive oxygen species activity and increasing malondialdehyde and superoxide dismutase activity. MMO improved mitochondrial dysfunction by decreasing the mitochondrial membrane potential and increasing the activities of Na^+^/K^+^-ATPase and Ca^2+^/Mg^2+^-ATPase. In addition, MMO inhibited the activation of cell death-related pathways, based on reduced p-JNK, Bax expression, tumor necrosis factor-α, caspase-9, and caspase-3 activity in the NAFLD model cells, and Bcl-2 expression was enhanced in the NAFLD model cells compared with the control group. These findings indicate that MMO have antioxidant and anti-apoptotic effects on NAFLD model cells and may thus exert protective effects against NAFLD.

## 1. Introduction

Non-alcoholic fatty liver disease (NAFLD), characterized by the excessive accumulation of fat in the liver, is the most common liver disease in China [1]. Excessive fat accumulation leads to obesity and an increased risk for many metabolic diseases, including non-alcoholic fatty liver disease (NAFLD), insulin resistance, hypertension, and hyperlipidemia [2,3,4]. NAFLD comprises various clinical conditions ranging from steatosis to non-alcoholic steatohepatitis, fibrosis, cirrhosis, and hepatocellular carcinoma [5]. Therefore, controlling NAFLD could help to reduce the risk of various liver diseases. Currently, however, no food and drug treatments are available for NAFLD. The discovery of functional foods or food-sourced functional factors could provide an efficient approach to prevent NAFLD [6,7].

Marine organisms such as shellfish are a rich source of structurally diverse bioactive nitrogenous components [8,9]. Polysaccharides obtained from marine origins are one type of these biochemical compounds that have several important properties, such as immunomodulatory, anticoagulant, antithrombotic, antitumor and cancer preventive, anti-inflammatory, antibiotic, antioxidant, antilipidaemic, and hypoglycaemic activities [10]. Some of these bioactive compounds are novel proteins and chemical entities which can be applied to drug discovery [11]. The skeletal organic matrix proteins derived from marine calcifiers, for example, have been concentrated on biomedical applications such as the identification of growth inducing proteins that can be used for bone regeneration [11,12].

Because they are a rich source of protein, marine organisms are ideal starting materials for the generation of protein-derived bioactive peptides.

*Meretrix meretrix* widely distributes in the coastal areas of China and is an important economic mudflat shellfish resource. It tastes delicious and contains carbohydrates, protein, and vitamins. It also exhibits medicinal activities, such as anticancer [13], antioxidant [14], and antiaging effects [15]. Management of functional food use is considered beneficial for treating NAFLD [16].

In a previous study, *Meretrix meretrix* was hydrolyzed by pepsin, alkaline, protease, neutral protease, papain, and trypsin. The best hydrolysis process was determined by orthogonal testing, including temperature, time, dose, pH, and feed solution proportion. The best hydrolysis conditions based on an index of triglyceride content were a temperature of 40 °C, time of 8 h, alcalase enzyme dosage of 1000 U/g, pH 9.5, and a solid-liquid ratio of 1:2. The amino acid sequence of the derived *Meretrix meretrix* oligopeptides (MMO) was Gln-Leu-Asn-Trp-Asp. Human Chang liver cells were treated with 15 μg/mL palmitic acid for 48 h as a NAFLD cell model. Treatment with MMO significantly decreased the Oil Red O-stained material and the lipid contents of NAFLD model cells.

The functional value of MMO, however, has not been well evaluated, and, to our knowledge, no studies of the effects of MMO on NAFLD have been published. Therefore, the objective of this study was to investigate the protective effect of MMO on NAFLD using a Human Chang liver cell model.

## 2. Materials and Methods

### 2.1. Chemicals and Reagents

Anti-β-Actin antibody and other chemicals were high-grade products from Sigma (Shanghai, China). Antibodies against Bcl-2, Bax, and caspase-9 were obtained from Beijing ZSGB Biotechnology Co., Ltd. (Shanghai, China). Antibodies against Caspase-3, TNF-α, P-JNK, and JNK1/2/3 were purchased from ABGENT (San Diego, CA, USA). Cell culture materials were obtained from GBICO (Grand Island, NY, USA). A Cell cycle staining Kit and an Annexin V-FITC/PI Apoptosis Detection Kit were obtained from YTHX Biotechnology Co., Ltd. (Beijing, China). A BCA Protein Assay Kit was obtained from Beyotime Institute of Biotechnology (Nanjing, China). Minim ATP enzyme test kits (Na^+^-K^+^ and Ca^2+^-Mg^2+^, T-ATP enzyme) were obtained from Nanjing jiancheng Bioengineering Insitute (Nanjing, China). A CellTiter-Glo (CTG) luminescent assay kit was obtained from Promega (Fitchburg, MA, USA). ROS, MDA, and SOD detection kits were purchased from Nanjing Jiancheng Bioengineering Insitute (Nanjing, China).

### 2.2. Preparation of Meretrix meretrix Oligopeptides (MMO)

**Figure 1:**
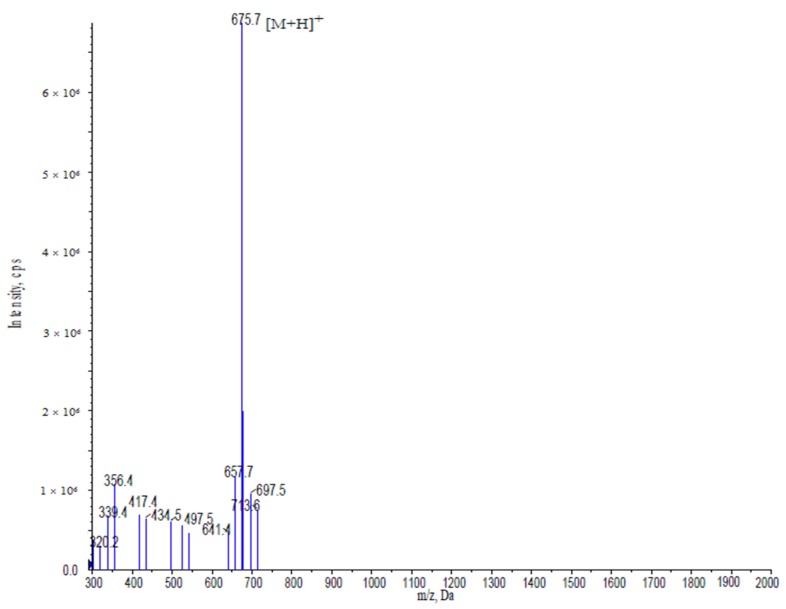
Mass spectrogram of *Meretrix meretrix* oligopeptides (MMO). MMO sequences were identified as Gln-Leu-Asn-Trp-Asp.

The *Meretrix meretrix* was hydrolyzed by pepsin, alkaline, protease, neutral protease, papain, and trypsin. The best hydrolysis process was determined by orthogonal testing, including temperature, time, dose, pH, and feed solution proportion. The best hydrolysis conditions based on an index of triglyceride content were a temperature of 40 °C, time of 8 h, alcalase enzyme dosage of 1000 U/g, pH 9.5, and a solid-liquid ratio of 1:2. *Meretrix meretrix* oligopeptides (MMO) was identified by a LTQ-Orbitrap mass spectrometer (Thermo, Waltham, MA, USA) coupled with an electrospray ionisation (ESI) source. The molecular mass of MMO was determined by the doubly charged (M + 2H)^+2^ state peak in the mass spectrum. MMO was sequenced on a Shimadzu PPSQ-31A automated gasphase sequencer (Shimadzu, Kyoto, Japan). It was dissolved in 20 µL of a 37% CH_3_CN (*v/v*) solution and applied to TFA-treated glass fiber membranes, precycled with Polybrene (Shimadzu, Kyoto, Japan). Data were recorded using the Shimadzu PPSQ-31A software. The amino acid sequence of the MMO was Gln-Leu-Asn-Trp-Asp, and its molecular mass was 675.7 Da (Figure 1).

### 2.3. Cell Culture and the NAFLD Model Cells

The human Chang Liver cells were purchased from cell center of Xiang Ya Hospital, Central South University. The cells were cultured in DMEM with high glucose and supplemented with 10% foetal bovine serum, 100 units/mL penicillin, 100 μg/mL streptomycin in a humidified incubator under 95% air, and 5% CO_2_ at 37 °C. Human Chang liver cells were used in our experiments after exposure to palmitic acid at a final concentration of 15 μg/mL for 48 h to induce an oerload of fatty acid as NAFLD model cells.

### 2.4. Cell Viability Assay

The number of viable cells in culture based on the quantitation of the ATP present produced by metabolically active cells were determined using the CellTiter-Glo (CTG) luminescent assay kit (Promega, Fitchburg, WI, USA), as per the manufacturer’s instructions. Briefly, the human Chang Liver cells were seeded in 96-well plates at 1 × 10^4^ cells per well. The cells were treated with 15 μg/mL palmitic acid for 48 h as NAFLD model cells. The control group, was the human Chang Liver cells incubated without MMO and palmitic acid. Forty-eight hours later, the NAFLD model cells were incubated with 10 and 20 mg/mL of MMO for 24 h. A volume of CTG reagent equal to the volume of the cell culture medium present in each well was added to each well. The generated luminescent signal was captured on a Multi-Detection Microplate Reader (Synergy HTX, BioTek Instrument, Inc., Winooski, VT, USA). The results were expressed as percentage of control.

### 2.5. ATPase Activities

The cells were treated with 15 μg/mL palmitic acid for 48 h as NAFLD model cells. The control group was the human Chang Liver cells incubated without MMO and palmitic acid. 48 h later, the NAFLD model cells were incubated with 10 and 20 mg/mL of MMO for 24 h. The activities of Na^+^/K^+^-ATPase and Ca^2+^/Mg^2+^-ATPase were measured as per the manufacturer’s instructions (Nanjing Jiancheng Bioengineering Institute, Nanjing, China).

### 2.6. Reactive Oxygen Species (ROS) Levels

Reactive oxygen species (ROS) levels were detected as per the manufacturer’s instructions (Nanjing Jiancheng Bioengineering Institute, Nanjing, China). Briefly, the human Chang Liver cells were seeded in 24-well plates at 5 × 10^4^ cells per well. The cells were treated with 15 μg/mL palmitic acid for 48 h as NAFLD model cells. The control group was the human Chang Liver cells incubated without MMO and palmitic acid. Forty-eight hours later, the NAFLD model cells were incubated with 10 and 20 mg/mL of MMO for 24 h. After MMO treatment, cells were incubated with PBS containing 20 µM DCF-DA at 37 °C for 30 min. The fluorescent intensity of DCF was detected by The Multi-Detection Microplate Reader (Synergy HTX, BioTek Instrument, Inc., Winooski, VT, USA) at an excitation wavelength of 480 nm and at an emission wavelength of 525 nm. Data were expressed as a relative percentage of DCF-fluorescence in the control cells.

### 2.7. Malondialdehyde (MDA) and Superoxide Dismutase Levels

The levels of MDA and Superoxide Dismutase were detected by using the MDA detection kit and Superoxide dismutase (SOD) kit (Nanjing Jiancheng Bioengineering Institute, Nanjing, China), respectively, according to the manufacture’s protocols. Shortly, the cells were seeded in 6-well plates at 5 × 10^6^ cells per well. The human Chang Liver cells were seeded in 24-well plates at 5 × 10^4^ cells per well. The cells were treated with 15 μg/mL palmitic acid for 48 h as NAFLD model cells. 48 h later, the NAFLD model cells were incubated with 10 and 20 mg/mL of MMO for 24 h. The optical density was read on a Multi-Detection Microplate Reader (Synergy HTX, BioTek Instrument, Inc., Winooski, VT, USA), and the protein concentrations were determined by a BCA Protein Assay Kit (Beyotime, Nanjing, China). Data were expressed as nmol/mg protein.

### 2.8. Cell Apoptosis Analysis

Apoptotic and necrotic cell death were evaluated as per the manual operation of an Annexin V Staining Kit (YTHX Biotechnology Co., Ltd., Beijing, China). In brief, the cells were treated with 15 μg/mL palmitic acid for 48 h as NAFLD model cells. 48 h later, the NAFLD model cells were incubated with 10 and 20 mg/mL of MMO for 24 h. The control group was the human Chang Liver cells incubated without MMO and palmitic acid. Subsequently, the cells were washed twice with cold phosphate-buffered saline (PBS) and then resuspended in 1× binding buffer at a concentration of 5 × 10^5^ cells/mL. 5 μL Annexin V and 10 μL PI were added to each tube. 15 min later, at room temperature and in the dark, 400 μL of 1× binding buffer was added to each tube. The samples were analyzed by a flow cytometry (Millipore/Guava Technologies, CA, USA).

### 2.9. Mitochondrial Membrane Potential (ΔΨm) Assay

JC-1 (5,50,6,60-tetra-chloro-1,10,3,30-tetra-ethylbenzimidalyl-carbocyanineiodide) staining was used to detect changes of ΔΨm. The cells were treated with 15 μg/mL palmitic acid for 48 h as NAFLD model cells. 48 h later, the NAFLD model cells were incubated with 10 and 20 mg/mL of MMO for 24 h. The control group was the human Chang Liver cells incubated without MMO and palmitic acid. After incubation, the collected cells were incubated with JC-1 2.5 µg/mL in 1 mL of PBS) for 30 min at 37 °C. The cells were then centrifuged (1000× *g*, 4 °C for 5 min), and the cells were washed twice with JC-1 staining buffer. Finally, the cells were resuspended in 200 µL of PBS. The cell-associated fluorescence was measured by flow cytometry.

### 2.10. Western Blot Analysis

The cells were treated with 15 μg/mL palmitic acid for 48 h as NAFLD model cells. 48 h later, the NAFLD model cells were incubated with 10 and 20 mg/mL of MMO for 24 h. The control group was the human Chang Liver cells incubated without MMO and palmitic acid. The harvested cells were washed with PBS and then lysed on ice for 30 min by RIPA lysis buffer. Subsequently, The cells were then centrifuged (1000× *g*, 4 °C for 5 min). The total protein concentration of the supernatant was quantified by a BCA protein assay kit, which was pre-heated for 10 min at 95 °C, then loaded on each channel, separated by SDS-PAGE, and electrophoretically transferred onto polyvinylidene difluoride membranes (PVDF). Finally, primary antibodies and secondary antibodies were utilized to hybridize corresponding proteins and visualized by enhanced chemiluminescence (ECL), as per the manufacturer’s recommendations.

### 2.11. Statistical Analysis

The results were expressed as the mean ± S.D. of at least three experiments performed using different in vitro cell preparations. Statistically significant differences were determined using a one-way ANOVA by SPSS 16 software. Statistical significance was set at *p* < 0.05.

## 3. Results and Discussion

### 3.1. Effect of MMO on the Viability of NAFLD Model Cells

**Figure 2:**
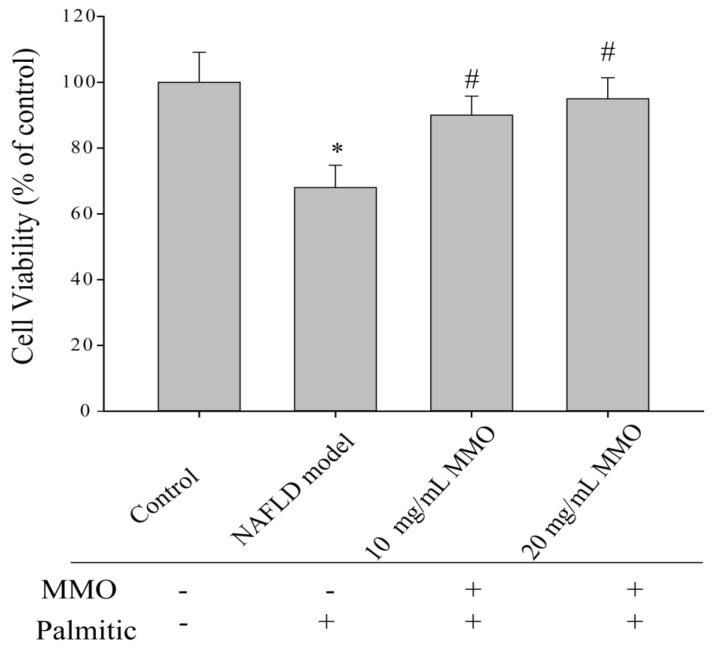
Effects of MMO on the cell viability of NAFLD model cells. The NAFLD model cells were treated with MMO (10 and 20 mg/mL) for 24 h, and cell viability was determined by CellTiter-Glo assay. Data are presented as mean ± S.D. for three independent experiments with triplicate determination. * *p* < 0.05 as compared with control. ^#^ *p* < 0.05 as compared with NAFLD model.

To determine the effect of MMO on the viability of NAFLD model cells, a CellTiter-Glo assay was performed (Figure 2). The viability of the NAFLD model cells was significantly reduced compared with control cells. Treatment of the NAFLD model cells with MMO for 24 h increased the viability compared with the untreated NAFLD model cells. This result was confirmed by flow cytometry, which demonstrated that 24-h MMO treatment suppressed apoptosis in the NAFLD model cells.

### 3.2. Effect of MMO on Apoptosis in NAFLD Model Cells

**Figure 3:**
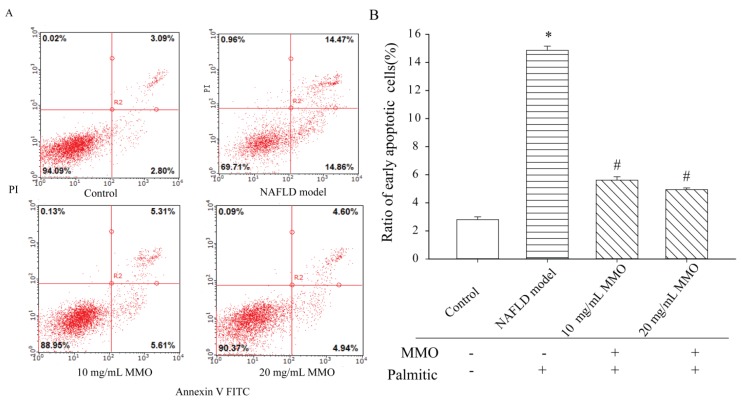
The apoptotic effects of MMO on nonalcoholic fatty liver disease (NAFLD) model cells. (**A**) The NAFLD model cells were treated with MMO (10 and 20 mg/mL) for 24 h, and cell apoptosis was detected by Annexin V-FITC/PI staining. LL represented normal cells (Annexin V^−^/P^−^), LR represented early apoptotic cells (Annexin V^+^/PI^−^), and UR represented late apoptotic ornecrosis cells (Annexin V^+^/PI^+^); (**B**) The percentage of apoptotic cells. Data are presented as mean ± S.D. for three independent experiments with triplicate determination.* *p* < 0.05 as compared with control. ^#^ *p* < 0.05 as compared with NAFLD model.

Apoptosis is an ATP-dependent process that helps to maintain tissue homeostasis under normal physiologic conditions [17]. In NAFLD, however, apoptosis is upregulated and triggers a profibrogenic and proinflammatory response from hepatocytes [18]. Apoptosis of NAFLD model cells treated with different concentrations of MMO for 24 h was evaluated based on Annexin V-FITC/PI staining. MMO treatment suppressed apoptosis in NAFLD model cells in a dose-dependent manner compared with untreated NAFLD model cells (Figure 3). MMO treatment at 10 and 20 mg/mL decreased the early apoptosis population in NAFLD model cells from 14.86% to 5.61% and 4.49%, respectively.

### 3.3. Effect of MMO on Oxidative Stress in NAFLD Model Cells

**Figure 4:**
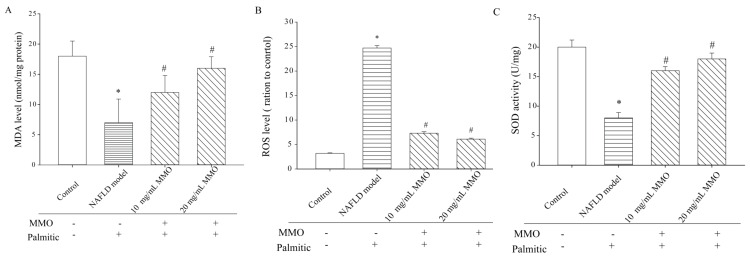
Effects of MMO on oxidative stress on NAFLD model cells. The NAFLD model cells were treated with MMO (10 and 20 mg/mL) for 24 h. (**A**) The level of MDA was detected by using the MDA detection kit; (**B**) the level of ROS was detected by using the ROS detection kit; (**C**) the level of SOD was detected by using the SOD detection kit. Data are presented as mean ± S.D. for three independent experiments with triplicate determination.* *p* < 0.05 as compared with control. ^#^ *p* < 0.05 as compared with NAFLD model.

Oxidative stress plays a central role in the pathogenesis of NAFLD due to the increased production of reactive oxygen species (ROS), which triggers the inflammatory response, and apoptosis, which causes lipid peroxidation [19,20]. Hepatic malondialdehyde (MDA) formation is commonly used as a biomarker of liver tissue damage [21]. Superoxide dismutase (SOD) the effects of MMO on NAFLD glutathione peroxidase, and catalase, the major antioxidant enzymes, are thought to act as the primary defense system against ROS generated during oxidative stress [22]. These antioxidant enzymes work cooperatively in the metabolic pathway of free radicals to avert oxidative damage [23]. To determine the effects of MMO on oxidative stress in NAFLD model cells, the levels of ROS, MDA, and SOD were measured (Figure 4). The levels of MDA and SOD (Figure 4C) were significantly reduced in the NAFLD model cells compared with the control cells. In the NAFLD model cells treated for 24 h with MMO, MDA, and SOD were significantly increased compared with the untreated NAFLD model cells (Figure 4A). The ROS (Figure 4B) levels, which were significantly increased in the NAFLD model cells at 24 h, were significantly reduced at 24 h after MMO treatment compared with the untreated NAFLD model cells. These results indicate that MMO alleviates oxidative stress and protects against NAFLD by its antioxidant activity.

### 3.4. Effect of MMO on Mitochondrial Function in NAFLD Model Cells

**Figure 5:**
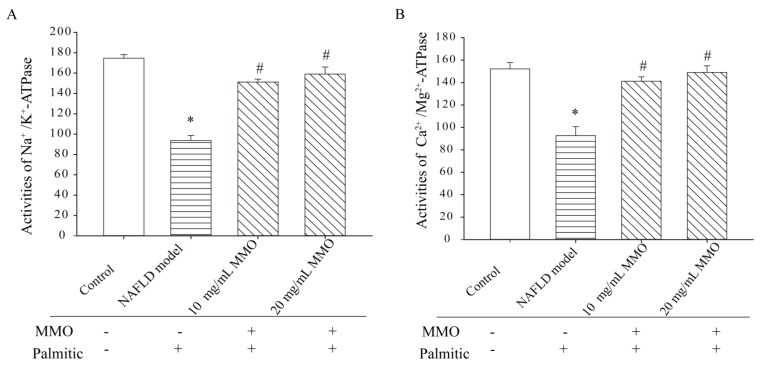
Effects of MMO on ATPase activities on NAFLD model cells. The NAFLD model cells were treated with MMO (10 and 20 mg/mL) for 24 h. (**A**) the activity of Na^+^/K^+^-ATPase was detected by using the Na^+^/K^+^-ATPase detection kit; (**B**) the activity of Ca^2+^/Mg^2+^-ATPase was detected by using the Ca^2+^/Mg^2+^-ATPase detection kit. Data are presented as mean ± S.D. for three independent experiments with triplicate determination.* *p* < 0.05 as compared with control. ^#^ *p* < 0.05 as compared with NAFLD model.

**Figure 6:**
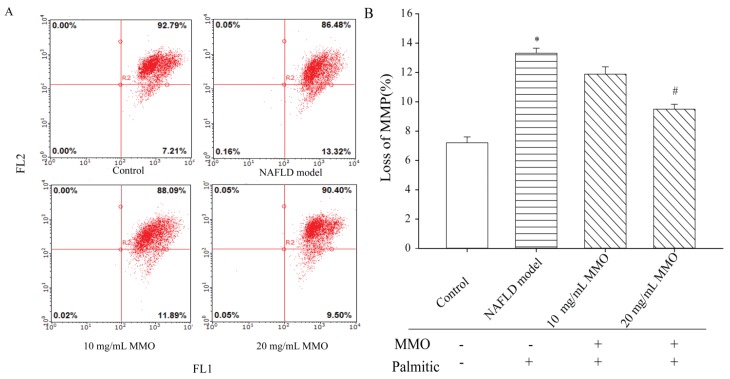
The effect of MMO on mitochondrial membrane potential on NAFLD model cells. (**A**) The NAFLD model cells were treated with MMO (10 and 20 mg/mL) for 24 h, and mitochondrial membrane potential was detected by the fluorescent dye JC-1 and analyzed by flow cytometry; (**B**) The percentage of loss of MMP. Data are presented as mean ± S.D. for three independent experiments with triplicate determination.* *p* < 0.05 as compared with control. ^#^ *p* < 0.05 as compared with NAFLD model.

Mitochondria produce the majority of cellular ATP through oxidative phosphorylation, and their capacity to do so is affected by many factors [24]. Na^+^/K^+^-ATPase and Ca^2+^/Mg^2+^-ATPase are a heterodimeric, transmembrane, ubiquitously present proteins that regulate substrate transportation, metabolic processes, ion homeostasis, neuronal signaling, and muscle contraction [25]. Na^+^/K^+^-ATPase and Ca^2+^/Mg^2+^-ATPase have key roles in maintaining intracellular calcium homeostasis [26]. Mitochondrial membrane potential is the major component of the proton motive force produced by the mitochondrial respiratory chain for ATP synthesis [27]. Therefore, the activities of Na^+^/K^+^-ATPase and Ca^2+^/Mg^2+^-ATPase and the mitochondrial membrane potential (MMP) were measured to determine whether the effects of MMO to suppress apoptosis of NAFLD model cells are due to the effects of MMO on mitochondrial function. The NAFLD model cells showed a significant loss of Na^+^/K^+^-ATPase and Ca^2+^/Mg^2+^-ATPase activities compared with the control cells (Figure 5), indicating that mitochondrial energy metabolism was blocked in the NAFLD model cells. Treatment of NAFLD model cells for 24 h with MMO at 10 mg/mL and 20 mg/mL led to increased Na^+^/K^+^-ATPase and Ca^2+^/Mg^2+^-ATPase activities in a dose-dependent manner. The ΔΨm of the NAFLD model cells (Figure 6) was significantly decreased by treatment with MMO for 24 h in a dose-dependent manner, compared with the untreated NAFLD model cells, but increased compared with control cells. Excessive ROS generation can result in mitochondrial dysfunction and induce apoptosis [28]. These results revealed that MMO promoted mitochondrial energy metabolism in the NAFLD model cells and contributed to normalize mitochondrial energy metabolism, suggesting that MMO improves mitochondrial dysfunction.

### 3.5. Effect of MMO on Alleviating Cell Death-Associated Protein Expression and Activity in NAFLD Model Cells

**Figure 7:**
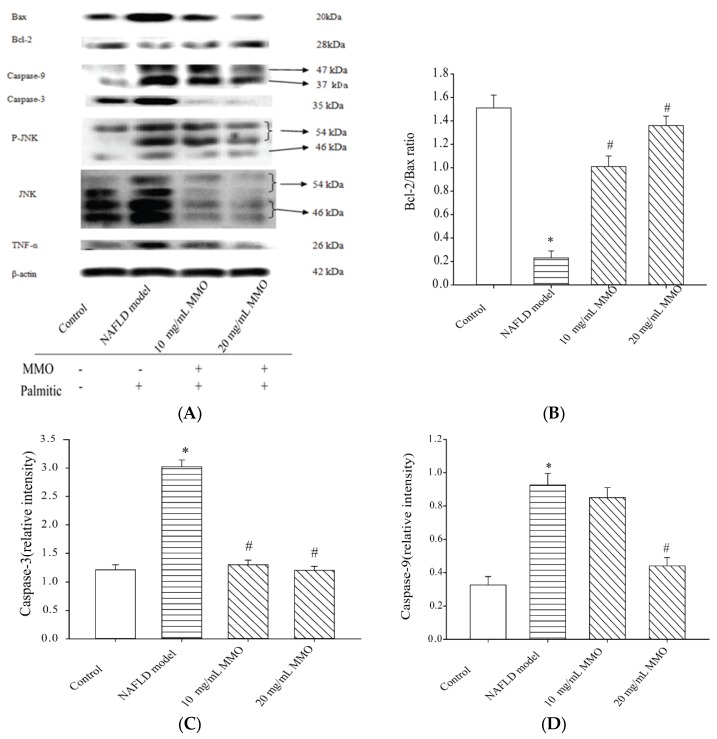

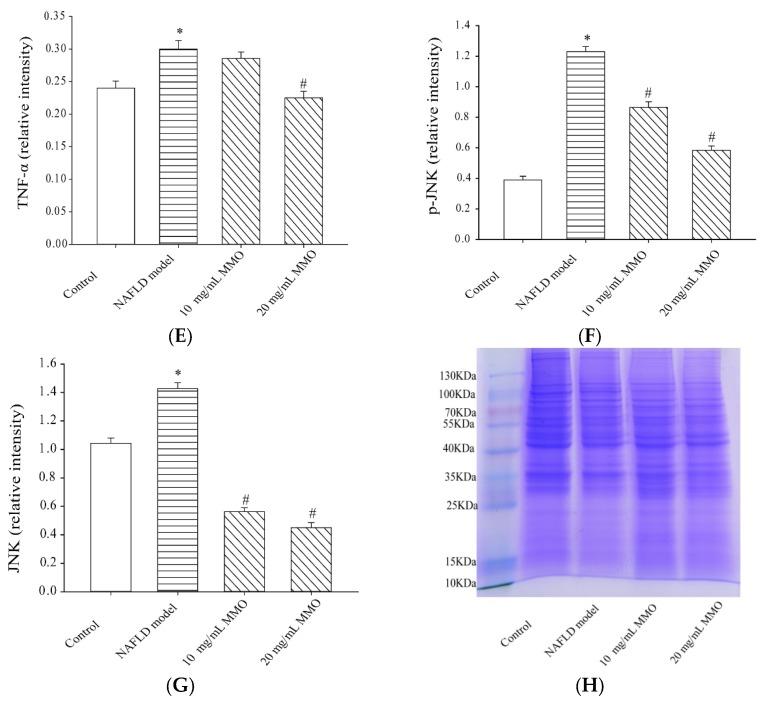
(**A**) The effects on Bax, Bcl-2, caspase-3, caspase-9, TNF-α, JNK, and p-JNK protein expression levels were detected by the Western blot method after exposure to MMO for 24 h; (**B**) Protein expression level of bax/bcl-2 was detected by Western blot after exposure to MMO; (**C**) Protein expression level of Caspase-3/β-actin was detected by Western blot after exposure to MMO; (**D**) Protein expression level of Caspase-9/β-actin was detected by Western blot after exposure to MMO; (**E**) Protein expression level of TNF-α/β-actin was detected by Western blot after exposure to MMO; (**F**) Protein expression level of p-JNK/β-actin was detected by Western blot after exposure to MMO; (**G**) Protein expression level of JNK/β-actin was detected by Western blot after exposure to MMO. β-actin was used as a control in all histograms; (**H**) SDS-PAGE image stained with Coomassie brilliant blue. Data are presented as mean ± S.D. for three independent experiments with triplicate determination.* *p* < 0.05 as compared with control. ^#^ *p* < 0.05 as compared with NAFLD model.

The above findings indicated that MMO alleviated cell death by suppressing apoptosis and alleviating oxidative stress in the NAFLD model cells. To determine whether the protective effects of MMO in the NAFLD model cells were associated with the cell death-related pathways leading to the recovery of normal hepatic function, we measured the expression of Bax and Bcl-2, JNK phosphorylation, caspase-3, caspase-9, and TNF-α activity. Bax expression (Figure 7A,B), JNK, and P-JNK (Figure 7A,C), TNF-α, caspase-9, and caspase-3 activity (Figure 7D) were enhanced in the NAFLD model cells, and Bcl-2 expression was decreased compared with the control cells. Current evidence suggests that JNK is activated and induces apoptosis in NAFLD model cells, which is relieved by MMO treatment. JNK activation induces mitochondrial dysfunction and apoptosis [29]. Cell apoptotic signaling is thought to be induced by oxidative stress [30]. Oxidative stress activates JNK signaling, which in turn induces apoptosis via Bax [30]. Apoptotic stimuli promote the translocation of mitochondrial Bax, which is a pro-apoptotic Bcl-2 family of proteins that decreases the mitochondrial membrane potential (MMP) and promotes the generation of ROS [31]. Apoptotic signaling is activated when Bax migrates to the mitochondrial surface [32], promotes cyt-c and AIF release from the mitochondria to the cytosol [33], cleaves procaspase-9 to caspase-9, and activates caspase-3 to promote cell death [34]. The present study demonstrated that the protective effects of MMO on NAFLD model cells may be due to the inhibition of pro-apoptotic pathway activation; antioxidant activity to decrease ROS, matrix metalloprotease, and TNF-α formation; and decreased JNK pathway activation.

Marine bioactive peptides obtained by hydrolysis processes have attracted heightened interest due to their health benefits. Marine bioactive peptides exert a wide range of biologic functions including antioxidant, antihypertensive, antimicrobial, antithrombotic, immunomodulatory, opioid agonistic, prebiotic, mineral binding, and hypocholesterolemic effects due to their structural properties, amino acid composition, and sequences [35,36,37,38]. The results of the present study demonstrated that MMO protected against NAFLD in a Chang cell model by its antioxidative and antiapoptotic effects. These findings provide new insight for potential health promotion and disease prevention. In this regard, MMO as a marine bioactive peptide may be a valuable functional food source that could be helpful in NAFLD therapy. Therefore, controlling NAFLD would reduce the risk of liver diseases such as cirrhosis and liver fibrosis as well as diabetes and metabolic syndromes [39].

## 4. Conclusions

In this study, the protective effects of MMO and the mechanism of action against NAFLD were evaluated in a NAFLD cell model. MMO alleviated oxidative stress, improved mitochondrial dysfunction, and inhibited activation of cell death-related pathways. These findings suggest that MMO has protective effects against NAFLD.

## References

[1] Kim M., Park Y.G., Lee H.-J., Lim S.J., Ahn H.R., Jung S.H., Nho C.W. (2015). Youngia denticulata attenuates diet-induced obesity-related metabolic dysfunctions by activating AMP-activated protein kinase and regulating lipid metabolism. J. Funct. Foods.

[2] Wakil S.J., Abuelheiga L.A. (2009). Fatty acid metabolism: Target for metabolic syndrome. J. Lipid Res..

[3] Begriche K., Igoudjil A., Pessayre D., Fromenty B. (2006). Mitochondrial dysfunction in NASH: Causes, consequences and possible means to prevent it. Mitochondrion.

[4] Neuschwander-Tetri B.A. (2010). Hepatic lipotoxicity and the pathogenesis of nonalcoholic steatohepatitis: The central role of nontriglyceride fatty acid metabolites. Hepatology.

[5] Meng F., Ning H., Sun Z., Huang F., Li Y., Chu X., Lu H., Sun C., Li S. (2015). Ursolic acid protects hepatocytes against lipotoxicity through activating autophagy via an AMPK pathway. J. Funct. Foods.

[6] Miller C.N., Yang J.Y., Avra T., Ambati S., Della-Fera M.A., Rayalam S., Baile C.A. (2015). A dietary phytochemical blend prevents liver damage associated with adipose tissue mobilization in ovariectomized rats. Obesity.

[7] Ross A.B., Godin J.P., Minehira K., Kirwan J.P. (2013). Increasing whole grain intake as part of prevention and treatment of nonalcoholic fatty liver disease. Int. J. Endocrinol..

[8] Harnedy P.A., FitzGerald R.J. (2012). Bioactive peptides from marine processing waste and shellfish: A review. J. Funct. Foods.

[9] Kim S.-K., Wijesekara I. (2010). Development and biological activities of marine-derived bioactive peptides: A review. J. Funct. Foods.

[10] De Jesus Raposo M.F., de Morais A.M., de Morais R.M. (2015). Marine polysaccharides from algae with potential biomedical applications. Mar. Drugs.

[11] Rahman M.A. (2016). An Overview of the Medical Applications of Marine Skeletal Matrix Proteins. Mar. Drugs.

[12] Green D.W., Padula M.P., Santos J., Chou J., Milthorpe B., Ben-Nissan B. (2013). A therapeutic potential for marine skeletal proteins in bone regeneration. Mar. Drugs.

[13] Wu J., Xu S., Luo S., Fang H. (2014). Antitumor activity of *Meretrix meretrix* glycopeptides MGP0501 in vivo. J. Nanchang Univ. (Nat. Sci.).

[14] Bai M.Y., He C., Wei H., Yang Q., Wang Y., Xiong H. (2013). Optimization of Enzymatic Hydrolysis of *Meretrix meretrix* Linnaeus. Protein and Analysis of the Antioxidant Activity of the Hydrolysates in vitro. Chin. Agric. Sci. Bull..

[15] Suleria H.A.R., Gobe G., Masci P., Osborne S.A. (2016). Marine bioactive compounds and health promoting perspectives; innovation pathways for drug discovery. Trends Food Sci. Technol..

[16] Ibrahim S.H., Kohli R., Gores G.J. (2011). Mechanisms of lipotoxicity in NAFLD and clinical implications. J. Pediatr. Gastroenterol. Nutr..

[17] Fealy C.E., Haus J.M., Solomon T.P.J., Pagadala M., Flask C.A., McCullough A.J., Kirwan J.P. (2012). Short-term exercise reduces markers of hepatocyte apoptosis in nonalcoholic fatty liver disease. J. Appl. Physiol..

[18] Jou J., Choi S.S., Diehl A.M. (2008). Mechanisms of disease progression in nonalcoholic fatty liver disease. Semin. Liver Dis..

[19] Houstis N., Rosen E.D., Lander E.S. (2006). Reactive oxygen species have a causal role in multiple forms of insulin resistance. Nature.

[20] Videla L.A., Rodrigo R., Araya J., Poniachik J. (2006). Insulin resistance and oxidative stress interdependency in non-alcoholic fatty liver disease. Trends Mol. Med..

[21] Mansour M.A. (2000). Protective effects of thymoquinone and desferrioxamine against hepatotoxicity of carbon tetrachloride in mice. Life Sci..

[22] Inal M.E., Kanbak G., Sunal E. (2001). Antioxidant enzyme activities and malondialdehyde levels related to aging. Clin. Chim. Acta.

[23] Yan Z., Fan R., Yin S., Zhao X., Liu J., Li L., Zhang W., Ge L. (2015). Protective effects of Ginkgo biloba leaf polysaccharide on nonalcoholic fatty liver disease and its mechanisms. Int. J. Biol. Macromol..

[24] Chad A., Galloway H.L., Paul S. (2014). Brookes, and Yisang Yoon Decreasing mitochondrial fission alleviates hepatic steatosis in a murine model of nonalcoholic fatty liver disease. Am. J. Physiol.-Gastrointest. Liver Physiol..

[25] Wang H.Y., O’Doherty G.A. (2012). Modulators of Na/K-ATPase: A patent review. Expert Opin. Ther. Pat..

[26] Singh P., Kesharwani R.K., Misra K., Rizvi S.I. (2015). The modulation of erythrocyte Na(+)/K(+)-ATPase activity by curcumin. J. Adv. Res..

[27] Serviddio G., Bellanti F., Giudetti A.M., Gnoni G.V., Petrella A., Tamborra R., Romano A.D., Rollo T., Vendemiale G., Altomare E. (2010). A silybin-phospholipid complex prevents mitochondrial dysfunction in a rodent model of nonalcoholic steatohepatitis. J. Pharmacol. Exp. Ther..

[28] Liang H.L., Sedlic F., Bosnjak Z., Nilakantan V. (2010). SOD1 and MitoTEMPO partially prevent mitochondrial permeability transition pore opening, necrosis, and mitochondrial apoptosis after ATP depletion recovery. Free Radic. Biol. Med..

[29] Xu Y., Huang S., Liu Z.G., Han J. (2006). Poly(ADP-ribose) polymerase-1 signaling to mitochondria in necrotic cell death requires RIP1/TRAF2-mediated JNK1 activation. J. Biol. Chem..

[30] Levrand S., Vannay-Bouchiche C., Pesse B., Pacher P., Feihl F., Waeber B., Liaudet L. (2006). Peroxynitrite is a major trigger of cardiomyocyte apoptosis in vitro and in vivo. Free Radic. Biol. Med..

[31] Guha P., Dey A., Sen R., Chatterjee M., Chattopadhyay S., Bandyopadhyay S.K. (2011). Intracellular GSH depletion triggered mitochondrial Bax translocation to accomplish resveratrol-induced apoptosis in the U937 cell line. J. Pharmacol. Exp. Ther..

[32] Cory S., Adams J.M. (2002). The Bcl2 family: Regulators of the cellular life-or-death switch. Nat. Rev. Cancer.

[33] Scarabelli T.M., Knight R., Stephanou A., Townsend P., Chen-Scarabelli C., Lawrence K., Gottlieb R., Latchman D., Narula J. (2006). Clinical Implications of Apoptosis in Ischemic Myocardium. Curr. Probl. Cardiol..

[34] Li P., Nijhawan D., Budihardjo I., Srinivasula S.M., Ahmad M., Alnemri E.S., Wang X. (1997). Cytochrome c and dATP-Dependent Formation of Apaf-1/Caspase-9 Complex Initiates an Apoptotic Protease Cascade. Cell.

[35] Ngo D.H., Vo T.S., Ngo D.N., Wijesekara I., Kim S.K. (2012). Biological activities and potential health benefits of bioactive peptides derived from marine organisms. Int. J. Biol. Macromol..

[36] Kim S.K., Mendis E. (2006). Bioactive compounds from marine processing byproducts—A review. Food Res. Int..

[37] Najafian L., Babji A.S. (2012). A review of fish-derived antioxidant and antimicrobial peptides: Their production, assessment, and applications. Peptides.

[38] Betoret E., Betoret N., Vidal D., Fito P. (2011). Functional foods development: Trends and technologies. Trends Food Sci. Technol..

[39] Jung J.-H., Kim H.-S. (2013). The inhibitory effect of black soybean on hepatic cholesterol accumulation in high cholesterol and high fat diet-induced non-alcoholic fatty liver disease. Food Chem. Toxicol..

